# Venæsection in Inflammatory Affections of the Bladder

**Published:** 1813-06

**Authors:** Robert Semple

**Affiliations:** Member of the Royal College of Surgeons in London. 85, High-street, Islington


*To the Editors of the Medical and Physical Journal.*

GENTLEMEN,

GENTLEMEN,

IN looking over your Number for April, I find a query by Mr. Weatherhead, respecting the propriety of Venæsection *ad deliquium,* in inflammatory affections of the neck of of the bladder. A case of this kind occurs to my recollection, which happened on-board a ship of war, where there were evident symptoms of inflammation at the neck of the bladder. It was the case of a seaman, who had been in the habit of getting frequently intoxicated, and thereafter exposing himself to cold and wet. Being once in the state above described, and having slept for several hours on the damp deck of the vessel, he was carried below to his hammock by his comrades, but he could get no sleep, was restless and disturbed, and had an urgent desire to void urine, but without the power of doing so. The pain at the neck of the bladder was excruciating, and he became slightly delirious. In this state he was brought to me. The distended bladder filled the whole of the hypogastric region, and was very painful to the touch. I immediately endeavored to introduce a catheter into the bladder; I however could not pass the sphincter; and, although I proceeded with the greatest caution, yet the patient complained of much pain where the instrument stopt. Fearful of injuring the parts, I withdrew the catheter, and ordered a warm bath to be got ready, which was soon done, and I put my patient into it, and after a little time I tried the operation again, but without effect. I then tried to pass a small bougie, but with a similar result. I moreover administered the usual remedies recommended for spasmodic affections of that organ, which likewise failed. There was then no resource, but either to puncture the bladder or try the effects of copious venæsection, as the symptoms were urgent and alarming. I determined to adopt the latter practice, and that the end might be more fully answered, I got the man supported in an upright posture in the bath, and made a large orifice in one of the veins of the arm, and allowed the blood to flow till syncope was induced; and then, with the greatest ease imaginable, I passed a catheter into the bladder, by which an incredible quantity of urine was discharged, and the patient when he revived expressed himself "in heaven." I allowed an elastic catheter to remain in the bladder nearly two days, and with the antiphlogistic regimen the man finally got well. Perhaps this case may not agree with the one Mr. Weatherhead means; but if you consider it worth insertion in your Journal, it is very much at your service.

I have the honor to be, Gentlemen,

Your obedient Servant,

ROBERT SEMPLE,

Member of the Royal College of Surgeons in London.

85, *High-street, Islington,*

*April* 5, 1813.

